# Dr. William Warren Potter

**Published:** 1911-04

**Authors:** 


### OBITUARY

Dr. William Warren Potter, editor of the Buffalo Medical Journal, died at Buffalo, N. Y. on Tuesday, March 14, 1911, after an illness of a few weeks, aged 72 years.

Dr. Potter's life was one of activity and his high professional attainments made him prominent in the fields of medicine, surgery and literature, and the influence of his personality on medical literature will be a lasting one. His career was foreshadowed as to its success and the line of achievement by his lineage, since his father, his grandfather, and his greatgrandfather, not to mention collateral issue, were all distinguished physicians.

William Warren Potter was born at Strykersville, N. Y., December 31, 1838, and was the son of Dr. Lindorf and Mary Green (Blanchard) Potter. His academic education was received at Arcade Seminary, N. Y., and at Genesee Seminary and College, Lima, N. Y. He then entered the medical department of the University of Buffalo from which he received his degree in 1859. Upon the completion of his medical studies he formed a partnership with his uncle, Dr. Milton E. Potter, of Cowlesville, N. Y., and began the practice of medicine.

At the beginning of the civil war, Dr. Potter offered his services to the government. He passed the examination of the army board at Albany a few days after Fort Sumter was taken and in the summer of 1861 was commissioned assistant surgeon of the 49th regiment, New York volunteers; he served in the army of the Potomac under McClellan during the Peninsula and Antietam campaigns and under Burnside in the Fredericksburg disaster; was left in charge of wounded soldiers while army was retreating to Harrison's Landing; was captured by confederates June 30, 1862, and had an interesting interview with Stonewall Jackson. He was confined in Libby Prison, but was soon exchanged, and returned to his regiment. In December, 1862, he was promoted to the rank of surgeon and served with the 57th regiment, N. Y., volunteers, during the Chancellorsville and Gettysburg campaigns. Soon after the battle of Gettysburg he was assigned to the charge of the 1st division hospital, 2d army corps, and continued on that duty until mustered out of service with his regiment at the close of the war. He was brevetted by the President of the United States, for faithful and meritorious service, lieutenant colonel of United states volunteers, and by the governor of New York state, for like reasons, lieutenant colonel of New York volunteers.

Returning to civil life Dr. Potter practised his profession at Mount Morris, and at Batavia, N. Y., for a time, but soon returned to Buffalo, limiting his practice to diseases of women. In July, 1888, he became editor of the BUFFALO MEDICAL JOURNAL and shortly thereafter owner as well. He was one of the founders of the American Association of Obstetricians and Gynecologists, of which he was secretary and editor of its transactions from its organisation in 1888. He was a frequent contributor to medical literature and often was invited to address the more important medical bodies of the country.

One of the most valuable services rendered by Dr. Potter to the medical profession was his participation in the struggle for the establishment of the New York State Medical Examining Board. During those days of factional opposition, Dr. Potter spent much time at Albany lending his aid in favor of the bill which was finally passed by the legislature, signed by the Governor, and went into effect September 1, 1891, serving as the basis of the present system of state medical examination. Dr. Potter was appointed examiner in obstetrics and gynecology of the board, and since 1897 has also been president.

Dr. Potter was a member of the American Medical Association (chairman of the Section of the Diseases of Women, (1890); Medical Society State of New York (president, 1891); Medical Society County of Erie (president, 1893); Buffalo Medical and Surgical Association (president 1886); Buffalo Obstetrical Society, 1884-1886; president Section of Gynecology and Abdominal Surgery, First Pan-American Medical Congress, 1893; president of the National Confederation of Medical Examining and Licensing Boards, 1895-1899; secretary of the American Association of Obstetricians and Gynecologists since 1888; examiner in obstetrics and gynecology, New York State Board of Medical Examiners, and president since 1897.

Among hospital appointments held was that of consulting surgeon to the Buffalo General Hospital. Dr. Potter was a companion of the Military Order of the Loyal Legion of the United States; a member of Bidwell-Wilkinson Post, G. A. R.; and of the Army and Navy Club, New York.

Dr. Potter married March 23, 1859, Emily A. Bostwick, of Lancaster, N. Y., who died in 1906. He is survived by two daughters, Mrs. B. G. Tallman and Miss Alice Blanchard Potter of Buffalo. Dr. Potter's only son, Dr. Frank Hamilton Potter, died in 1891.

The funeral services were held at 238 Delaware avenue on Thursday, March 16, Rev. Cameron J. Davis, rector of Trinity

Church, officiating. Representatives from many societies and organisations of which Dr. Potter was a member attended. Dr. Augustus S. Downing, first assistant commissioner of education, represented the State Education Department; the members of the State Board of Medical Examiners in attendance were Dr. Frank W. Adriance of Elmira, Dr. Ralph H. Williams of Rochester, Dr. Lee H. Smith of Buffalo and Dr. M. J. Lewi of New York, the secretary of the Board. The Medical Society of the County of Erie was represented by its president, Dr. D. V. McClure and a committee of its members. There were present also members of the Military Order of the Loyal Legion and of Bidwell-Wilkinson Post, G. A. R.

The floral tributes were beautiful, the insignia of the Loyal Legion with its mass of color occupying a prominent place among the remembrances from other organisations and friends.

The bearers were Henry H. Seymour, Edmund S. Wheeler, Dr. Lee H. Smith, Dr. Herman E. Hayd, Dr. F. Park Lewis, Dr. Charles G. Stockton, Dr. Marshall Clinton and Dr. L. Burrows, Jr.

